# AMPK as a Potential Therapeutic Target for Intervertebral Disc Degeneration

**DOI:** 10.3389/fmolb.2021.789087

**Published:** 2021-12-08

**Authors:** Zhen Wang, Jianxiong Shen, Erwei Feng, Yang Jiao

**Affiliations:** Department of Orthopedics, Peking Union Medical College Hospital, Peking Union Medical College and Chinese Academy of Medical Sciences, Beijing, China

**Keywords:** low back pain, intervertebral disc degeneration, nucleus pulposus, AMPK, targeted therapy

## Abstract

As the principal reason for low back pain, intervertebral disc degeneration (IDD) affects the health of people around the world regardless of race or region. Degenerative discs display a series of characteristic pathological changes, including cell apoptosis, senescence, remodeling of extracellular matrix, oxidative stress and inflammatory local microenvironment. As a serine/threonine-protein kinase in eukaryocytes, AMP-activated protein kinase (AMPK) is involved in various cellular processes through the modulation of cell metabolism and energy balance. Recent studies have shown the abnormal activity of AMPK in degenerative disc cells. Besides, AMPK regulates multiple crucial biological behaviors in IDD. In this review, we summarize the pathophysiologic changes of IDD and activation process of AMPK. We also attempt to generalize the role of AMPK in the pathogenesis of IDD. Moreover, therapies targeting AMPK in alleviating IDD are analyzed, for better insight into the potential of AMPK as a therapeutic target.

## Introduction

Low back pain (LBP) is one of the most common complaints in the clinics of orthopedics. With a lifetime prevalence of more than 80%, LBP strikingly affects people’s daily life (Walker, 2000). As estimated, approximately 10% of the population affected turns out to be chronic and disabled, which brings about serious socio-economic burden (Urban and Roberts, 2003; Freburger et al., 2009). Despite of the fact that LBP is a multifactorial disease with complicated clinical manifestations, intervertebral disc (IVD) degeneration (IDD) is widely perceived as the major cause (Luoma et al., 2000). In the very initial stage, IDD is always asymptomatic or mildly symptomatic. However, with the course advancing, IVD herniation, spinal stenosis as well as spinal instability develop (Risbud and Shapiro, 2014). In other words, IDD serves as the pathological basis of degenerative spinal disorders. Aforementioned diseases bring about not only local pain, both also neurological symptoms, such as neuropathic pain, sensory deficit, hypodynamia (Yurube et al., 2020). Both the recurrent LBP and the deterioration of neurological function account for the disability induced by IDD. Currently, a variety of treatments have been prescribed for patients with discogenic LBP, including medication and surgery (Knezevic et al., 2017). Despite the complete removal of degenerative disc achieved by discectomy, approximately 30% of patients suffer from post spinal surgery syndrome (Palmer et al., 2019). As for conservative therapy, medicines available now are mainly aimed at relieving pain and improving the symptoms of nerve injury. Yet very few drugs approved target the pathogenesis of IDD and rescue the diseased IVD. Therefore, efforts should be devoted to the exploration of novel targeted drugs for IDD.

As an evolutionarily highly conserved serine/threonine-protein kinase, adenosine monophosphate (AMP) activated protein kinase (AMPK) is ubiquitously expressed in eukaryocytes (Hardie et al., 2012; Hardie, 2014). Previous study has identified the heterotrimeric complex structure of AMPK. Specifically, AMPK consists of a catalytic α subunit and two regulatory β- and γ-subunits. Each subunit has several isoforms, which are expressed in a tissue specific manner (Ross et al., 2016). As a critical intracellular energy sensor, the activity of AMPK is modulated by energy disequilibrium induced by various metabolic stresses, such as hypoxia, disturbance of blood supply, and deficiency of glucose (Salt et al., 1998; Marsin et al., 2000; Russell et al., 2004; Hardie, 2007; Dengler, 2020). Moreover, certain physiological factors, such as exercise and muscle contraction, may also serve as activators of AMPK (Vavvas et al., 1997; Hayashi et al., 1998; Musi et al., 2001; Ruderman et al., 2003). Once activated, AMPK participates in a wide range of cellular biological behaviors, including glycolipid metabolism, energy equilibrium, autophagy, etc., (Saikia and Joseph, 2021; Trefts and Shaw, 2021; Mihaylova and Shaw, 2011; Liang et al., 2007; Angin et al., 2016). Apart from the classical regulatory role of AMPK in cellular metabolism and energy balance, accumulating evidence indicates the involvement of AMPK in the pathogenesis of IDD (Chen et al., 2015; Jiang et al., 2015). In human degenerative IVD, an activation of AMPK signaling pathway has been observed (Yang et al., 2019). In contrast, an inhibition of AMPK phosphorylation has been confirmed in both human and rat cell models for IDD (Song et al., 2018; Liu et al., 2019b). Moreover, various AMPK activators, such as the illustrious metformin and resveratrol, have been proven protective in IDD, revealing AMPK as a promising target for the treatment of IDD (Chen et al., 2016; Wang et al., 2016). Herein, we comprehensively review the role of AMPK in IDD, to gain a better understanding of its therapeutic potential.

## Structure of Intervertebral Disc

**FIGURE 1:**
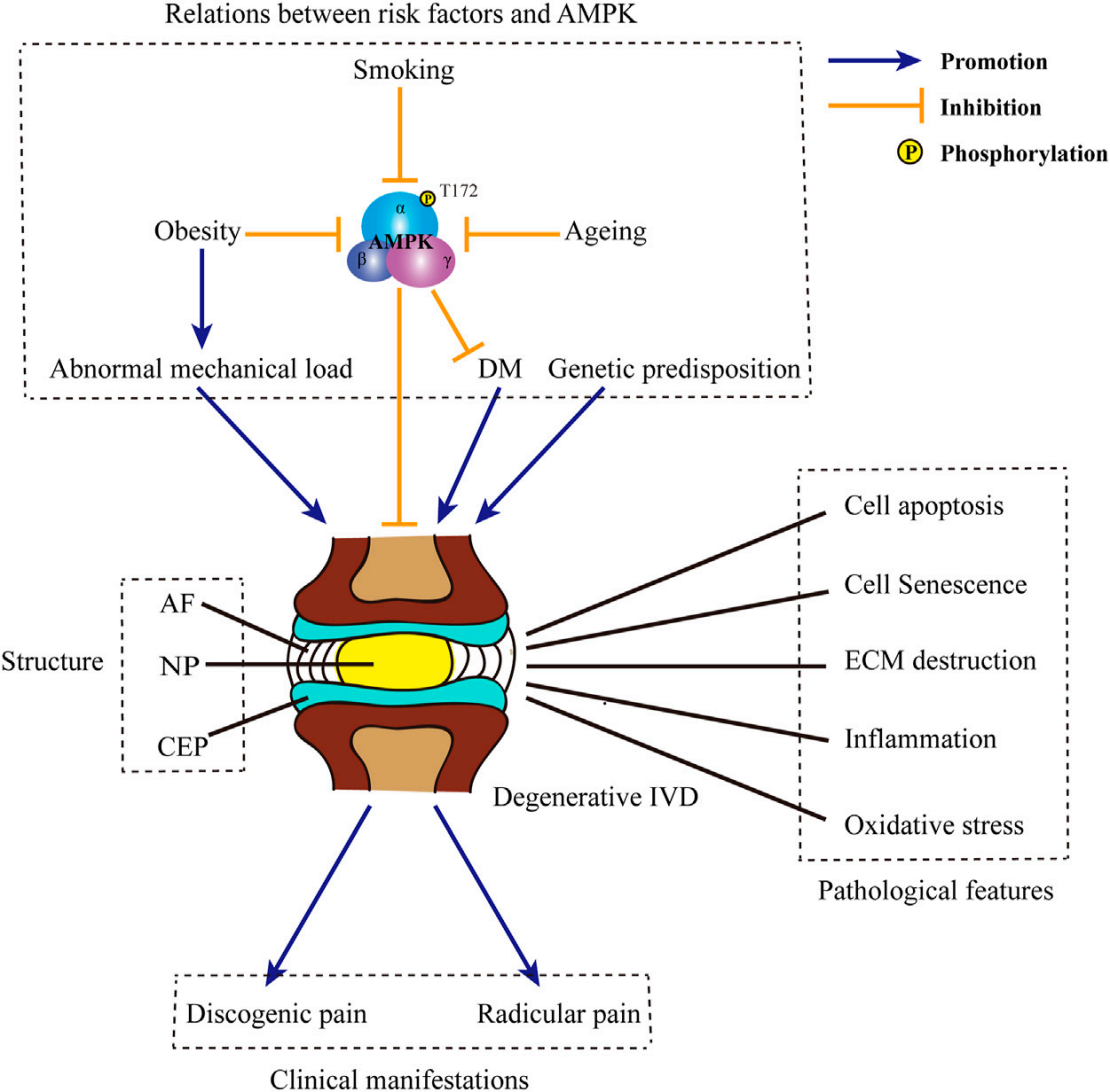
Structure of IVD and the pathogenesis of IDD. IVD is composed of three main parts: the core NP, the peripheral AF and CEP located in the top and bottom. Causative factors for IDD include obesity, ageing, smoking, DM, abnormal mechanical load, genetic predisposition, etc. Some of those factors, such as obesity, ageing and smoking may negatively regulate the phosphorylation of AMPK and inhibit AMPK activity. Besides, inactivation of AMPK may induce the occurrence of DM. Typical pathological features in degenerative IVD includes cell apoptosis and senescence, ECM destruction, inflammation, oxidative stress, etc. The progression of IDD may ultimately bring about discogenic pain and radicular pain. IVD: intervertebral disc; IDD: intervertebral disc degeneration; NP: nucleus pulposus; AF: annulus fibrosus; CEP: cartilage endplates; DM: diabetes mellitus; AMPK: AMP-activated protein kinase; ECM: extracellular matrix.

As the largest avascular origin in human body, IVD is a cartilaginous joint lying between adjacent vertebrae (Grunhagen et al., 2011). The IVD consists of three main components: the nucleus pulposus (NP), the annulus fibrosus (AF), and the cartilage endplates (CEP) (Figure 1). The gelatinous NP is located in the core of IVD. Apart from the well-known NP cells, NP tissue contains a wealth of extracellular matrix (ECM). The ECM surrounding the NP cells is rich of collagens and water-bound proteoglycans, giving the NP tissue high resilience and tensile strength to distribute stress in all directions (Chen S. et al., 2017). The AF around the NP is a fibrocartilaginous tissue composed of multi-storey collagen fibers. This laminated concentric structure helps to maintain the position and shape of NP and absorb mechanic shocks when NP is under pressure. Moreover, AF is involved in the energy metabolism of IVD. Through capillaries in peripheral tissues, AF cells located in the outer layer take in nutrients and release metabolic waste (Urban et al., 2004). The CEP is located in both the top and bottom of the IVD. As a layer of hyaline cartilage thinner than 1 mm, CEP withstands pressure and prevents the IVD from being overloaded (Moon et al., 2013). More importantly, CEP also plays a crucial role in the material exchange and nutrition supplement of IVD. Through the capillaries in CEP, most of the nutrients and substances required are diffused from consecutive vertebral bodies to the rest of IVD. Simultaneously, metabolites accumulated in the IVD are discharged (Grunhagen et al., 2006). This unique blood supply and substance exchange pathway composed by the CEP and outer annulus periphery bring about a hypoxia, low nutrition, and acidic microenvironment in IVD (Urban, 2002).

## Pathological Mechanism of Intervertebral Disc Degeneration

The etiology of IDD is multifactorial and not completely elucidated. To date, multiple factors have been recognized involved in the progression of IDD, such as ageing, smoking, diabetes mellitus (DM), obesity, abnormal mechanical load and genetic predisposition (Figure 1) (Kim et al., 2009; Le Maitre et al., 2007; Jeong et al., 2014; Desmoulin et al., 2020; Maclean et al., 2004; Walsh and Lotz, 2004; Wuertz et al., 2009; Kalb et al., 2012; Martirosyan et al., 2016; Alpantaki et al., 2019; Kakadiya et al., 2020; Livshits et al., 2001; Chen et al., 2018; Cannata et al., 2020). In response to those risk factors, various crucial pathological processes develop, including the loss of resident IVD cells, ECM remodeling, inflammation and oxidative stress (Figure 1) (Gruber and Hanley, 1998; Nasto et al., 2013; Suzuki et al., 2015; Lyu et al., 2021). Of above characteristic changes, cell loss is the most extensively studied one. In degenerative IVD, both cell apoptosis and cell senescence are prevalent (Roberts et al., 2006; Wang et al., 2008; Kim et al., 2009). Cell apoptosis directly decreases the number of viable cells; while cell senescence is accompanied by the reduction of functional cells. Moreover, cell apoptosis and senescence in IVD work cooperatively to bring about an impaired anabolism, in which a reduction in the synthesis of proteoglycans and the conversion of collagen II to collagen I are observed (Takaishi et al., 1997; Ngo et al., 2017). Meanwhile, senescent disc cells exhibit senescence-associated secretory phenotype and release various pivotal matrix proteases, such as matrix metalloproteases (MMPs) and a disintegrin-like and metalloprotease with thrombospondin type-1 motifs (ADAMTSs) (Le Maitre et al., 2007). The imbalance between the anabolism and catabolism of ECM brings about ECM destruction. The destruction of ECM further results in the dehydration of IVD, the loss of intervertebral height and the abnormal stress of IVD, which in turn accelerates the degeneration of IVD (Raj, 2008). During IDD, ECM destruction also enables the invasion of nerve fibers into the disc, which is an aneural organ in healthy state (Freemont et al., 2002; Melrose et al., 2002). The ingrowth of nerve fibers ultimately results in the occurrence of discogenic pain (Ohtori et al., 2018). Moreover, the disruption of ECM is also responsible for the rupture of AF and the simultaneous protrusion of NP (Guterl et al., 2013). Apart from matrix-degrading enzymes, a wide range of pro-inflammatory cytokines, including interleukin-1β (IL-1β) and tumor necrosis factor-α (TNF-α), are also secreted by the NP cells and AF cells (Shamji et al., 2010). Moreover, those resident disc cells also produce and release chemokines which recruit immune cells, such as macrophages, neutrophils and lymphocytes. The infiltration and activation of circulating immunocytes further enhance the inflammation reaction and bring about an inflammatory microenvironment inside and around the IVD (Risbud and Shapiro, 2014). Pro-inflammatory cytokines released by both resident disc cells and foreign immunocytes further exacerbate cell death and ECM destruction. In addition to discogenic LBP, IVD herniation and local inflammatory response in IDD may also contribute to the radicular pain in a mechanical and biochemical manner, respectively (Dower et al., 2019). Now that CEP is the principal source of nutrition for IVD, the destruction of CEP may also accelerate the process of IDD (Peng et al., 2001). Predisposing inducements, such as injury and ageing, lead to the cell loss and ECM remodeling in CEP (Ariga et al., 2001; Cinotti et al., 2005). ECM remodeling in degenerative CEP is characterized by the dramatic decline in the content of proteoglycans, especially chondroitin sulfate (Roberts et al., 1994). Changes in the contents of ECM ultimately lead to the calcification of CEP. Calcified ECM is accompanied with a decreased permeability and the destruction of capillaries (Jackson et al., 2011). Therefore, the calcification of CEP sets up obstacle for the exchange of nutrients and materials between vertebral bodies and IVD (Peng et al., 2001; Soukane et al., 2007). Insufficient supplement of nutrients and the accumulation of metabolites together bring about a tough microenvironment in degenerative IVD. This special local microenvironment may serve as a trigger for the overproduction of reactive oxygen species (ROS). Under this circumstance, numerous intrinsic or extrinsic inducers, such as pro-inflammatory cytokines, high glucose and mechanical strain, accelerates mitochondrial damage in resident disc cells and aggravates the excessive generation of ROS (Feng et al., 2017). Meanwhile, both the contents of enzymatic antioxidants and non-enzymatic antioxidants are reduced, leading to an impaired scavenging of ROS (Gruber et al., 2011; Hou et al., 2014). The abnormal accumulation of ROS results in oxidative injury in various intracellular substances and contributes to cell apoptosis, senescence and ECM remodeling in IVD (Feng et al., 2017).

## Activation of AMP-Activated Protein Kinase

**FIGURE 2:**
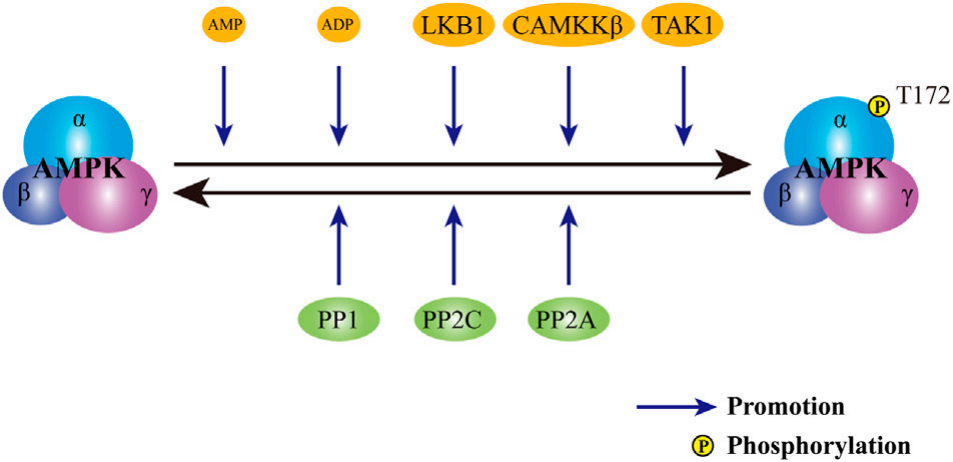
Regulation of AMPK activity. The activity of AMPK is subject to the control of both activators and inhibitors. Upon the stimuli of activators, including AMP, ADP, LKB1, CAMKKβ and TAK1, AMPK is phosphorylated and activated. In contrast, phosphatases, including PP2A, PP2C and PP1, dephosphorylates AMPK to avert persistent activation of AMPK. AMPK: AMP-activated protein kinase; AMP: adenosine monophosphate; ADP: adenosine diphosphate. LKB1: live kinase B1; CAMKKβ: calcium/calmodulin-dependent protein kinase kinase β; TAK1: transforming growth factor-β activated kinase 1; PP2A: protein phosphatase 2A; PP2C: protein phosphatase 2C; PP1: protein phosphatase 1.

To date, a variety of upstream signals have been identified as regulators of AMPK activation (Figure 2). As a critical metabolic switch, AMPK is sensitive to the change in the ratio of AMP to adenosine triphosphate (ATP). In physiological status, AMPK forms a complex with ATP and remains inactive. Upon the stimulation of energy deprivation or metabolic stress, intracellular level of AMP is elevated. Upregulated AMP binds to the γ-subunit of AMPK and triggers a conformational change (Moore et al., 1991). Altered conformation allows the phosphorylation of threonine 172 residue (Thr172) in the catalytic α-subunit by specialized kinases and the subsequent activation of AMPK (Hawley et al., 1996). Moreover, binding of AMP to the regulatory γ-subunit prolongs the activation of AMPK by the inhibition of dephosphorylation (Davies et al., 1995). Despite the inability to allosterically modulate AMPK, adenosine diphosphate (ADP) is also proven a trigger for the activation of AMPK at low concentrations (Carling et al., 1989). Apart from the first phosphorylation of AMPK mediated by AMP or ADP, a second phosphorylation is also required. As the major upstream kinase of AMPK in mammalian cells, live kinase B1 maintains the basal level of AMPK activity induced by AMP or ADP. Moreover, upon the increase in AMP:ATP ratio or ADP: ATP ratio, live kinase B1 further activates AMPK through the phosphorylation of Thr172 in α-subunit (Woods et al., 2003). In addition to AMP/ADP dependent canonical pathway, AMPK is also regulated by the change of intracellular calcium level. In response to the elevation in the concentration of calcium, the activity of calcium/calmodulin-dependent protein kinase kinase β is upregulated. Activated calcium/calmodulin-dependent protein kinase kinase β further phosphorylates Thr172 in α-subunit and leads to the activation of AMPK. Of note, the facilitation of calcium/calmodulin-dependent protein kinase kinase β on AMPK activity is independent on the phosphorylation of γ-subunit (Hawley et al., 2005; Hurley et al., 2005; Woods et al., 2005). As the third kinase for AMPK activation identified, transforming growth factor-β-activated kinase 1 can react to a wide range of stimuli, including starvation, bacterial infections and various extracellular cytokines, thereby directly phosphorylating the Thr172 in AMPK (Momcilovic et al., 2006; Neumann, 2018). In view of the crucial role of the Thr172 residue in AMPK activation, antagonists may target Thr172, thereby serving as a suppressor for AMPK. Through dephosphorylating Thr172 in α-subunit, various AMPK phosphatases, such as protein phosphatase 2A, protein phosphatase 2C, and protein phosphatase 1, avoid the continuous activation of AMPK (Salminen et al., 2016).

## AMP-Activated Protein Kinase in the Pathophysiology of Intervertebral Disc Degeneration

### Relationship Between AMP-Activated Protein Kinase and Risk Factors for Intervertebral Disc Degeneration

In view of its fundamental role in energy metabolism and energy balance, AMPK is in close touch with several predisposing factors for IDD (Figure 1). Actually, AMPK may function as an important intermediate between obesity and DM. Under obese circumstance, mitochondrial overheating is induced by oversupply of substrate to mitochondria in insulin-sensitive cells. Mitochondrial overheating brings about overproduction of ATP and the inhibition of AMPK (Ye, 2021). Meanwhile, obesity is often accompanied by the hyposecretion of adiponectin, an endogenous AMPK activator (Li et al., 2021). Through the modulation of AMPK activity, obesity results in the systemic insulin resistance and hyperglycemia (Ye, 2021). As reported, ageing process is always accompanied with the reduction of AMPK activation (Salminen et al., 2016). Insufficient activation of AMPK restricts organism to tackling with endogenous and exogenous stresses and leads to the imbalance of homeostasis. Therefore, decreased activity of AMPK may in turn accelerate the process of ageing. Of note, ageing is also a susceptible factor of insulin resistance (Vieira-Lara et al., 2021). Thus, it is speculated that the decline of AMPK activity during ageing may also participate in the occurrence and development of insulin resistance and DM.

In addition to above risk factors, smoking is also involved in the modulation of AMPK activity. As the organ directly exposed to cigarette smoking, lung is most affected. In smoking-induced mouse model of chronic obstructive pulmonary disease, the phosphorylation of AMPK was inhibited. Treatment enhancing AMPK phosphorylation alleviated inflammation and exerted protective effect in lung tissues (Jian et al., 2020). Although the role of smoking in IDD has been well-established, whether AMPK is involved in the facilitation of smoking on IDD remains further exploration.

### The Activity of AMP-Activated Protein Kinase in Intervertebral Disc Degeneration

To date, the activity of AMPK in degenerative IVD still remains controversial (Table 1). Wu and his colleagues reported an increase in the phosphorylation level of AMPK in degenerative IVDs in comparison to normal ones (Wu et al., 2017). Moreover, a recent study from Yang et al. confirmed that both the phosphorylation and the protein expression of AMPK were upregulated in rat degenerative IVD tissues and NP cells (Yang et al., 2019). However, in tert-Butyl hydroperoxide (TBHP)-exposed rat NP cells, which is a common *in vitro* cell model mimicking IDD, a slight but not statistically significant increase in the phosphorylation of AMPK was observed (Zhang et al., 2018). In contrast, a decrease in the phosphorylation of AMPK was identified in human NP cells stimulated by advanced glycation end products (AGEs), as well as in rat NP cells subject to lipopolysaccharide (Song et al., 2018; Liu et al., 2019b). The great differences between above studies indicate that there might be a dynamic change in the phosphorylation of AMPK during the course of IDD.

**TABLE 1:** The expression and activity of AMPK in IDD.

Human/Rat	Tissue/Cell	Disease model	AMPK expression	p-AMPK/AMPK ratio	References
Human	IVD tissues and AF cells	Degenerative disc disease	Not available	Upregulated	Wu et al. (2017)
Rat	IVD tissues and NP cells	Puncture-induced IDD	Upregulated	Upregulated	Yang et al. (2019)
Rat	NP cells	TBHP-treated NP cells	Not available	Unchanged	Zhang et al. (2018)
Human	NP cells	AGEs-treated NP cells	Not available	Downregulated	Song et al. (2018)
Rat	NP cells	LPS-treated NP cells	Not available	Downregulated	Liu et al. (2019b)

AMPK: AMP-activated protein kinase; IDD: intervertebral disc degeneration; p-AMPK: phosphorylated AMP-activated protein kinase; IVD: intervertebral disc; AF: annulus fibrosus; NP: nucleus pulposus; TBHP: tert-Butyl hydroperoxide; AGEs: advanced glycation end products; LPS: lipopolysaccharide.

### AMP-Activated Protein Kinase in the Pathogenesis of Intervertebral Disc Degeneration

**FIGURE 3:**
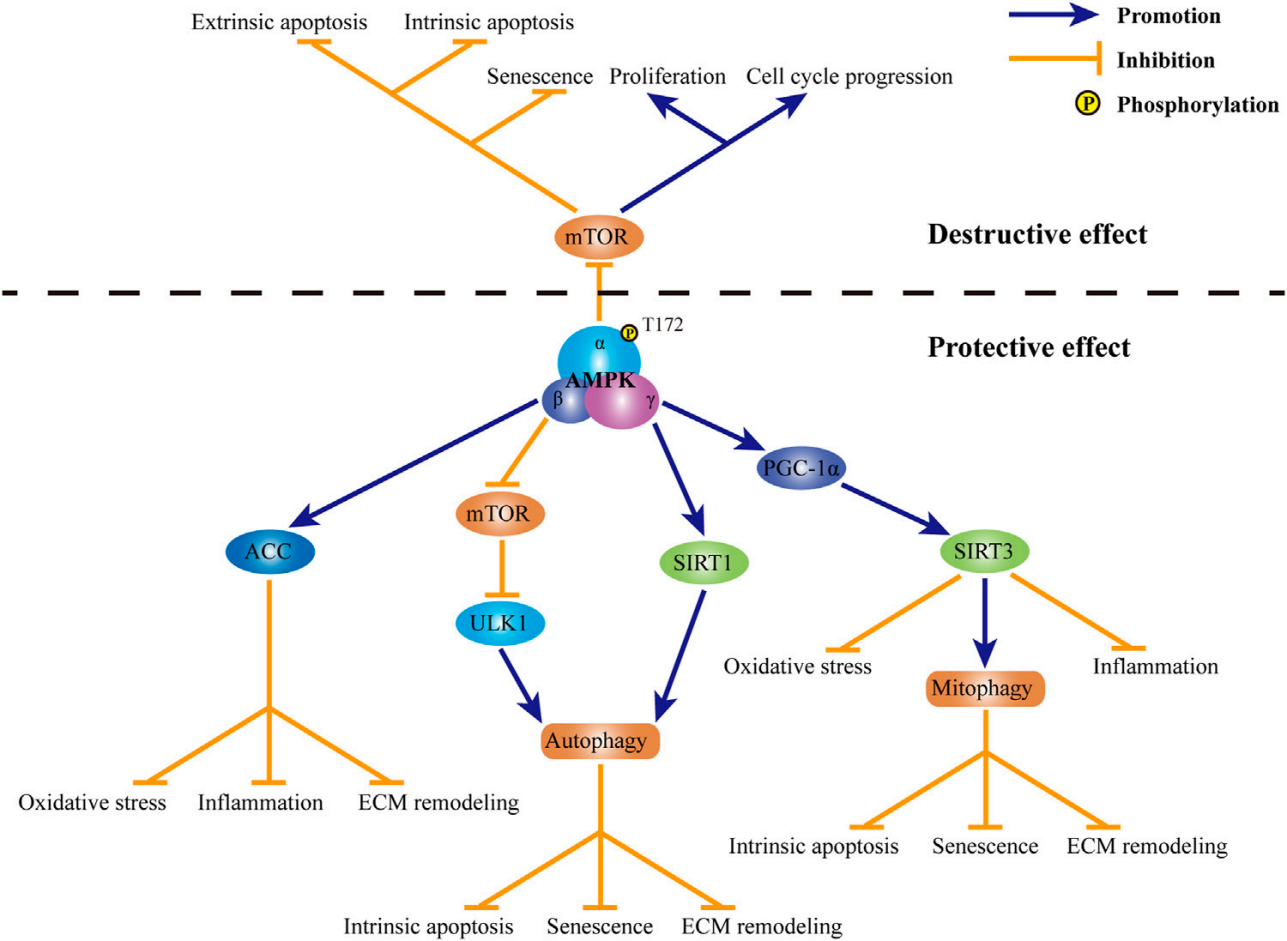
Role of AMPK in IDD. Currently, both destructive effect and protective effect of AMPK in IDD have been observed. Through the inhibiting of mTOR, AMPK promotes cell apoptosis along with cell senescence, suppresses cell proliferation and retards cell cycle progression in IVD cells. In contrast, mechanism for the protection of AMPK in IDD is complicated. Through the activation of ACC, AMPK suppresses oxidative stress, inflammation and ECM destruction in IVD cells. By modulating mTOR-ULK1 pathway, AMPK induces autophagy in IVD cells. SIRT1 is also involved in AMPK-mediated autophagy in IVD cells. Through the induction of autophagy, AMPK suppresses intrinsic apoptosis, senescence and ECM remodeling in IVD cells. Through the activation of PGC-1α and subsequent SIRT3, AMPK promotes mitophagy and inhibits oxidative stress and inflammation in IVD cells. By maintaining mitochondrial homeostasis, AMPK may protect IVD cells against intrinsic apoptosis, senescence and ECM destruction. AMPK: AMP-activated protein kinase; IDD: intervertebral disc degeneration; mTOR: mammalian target of rapamycin; IVD: intervertebral disc; ACC: acetyl-CoA carboxylase; ECM: extracellular matrix; ULK1: UNC-51-like kinase 1; SIRT1: sirtuin1; PGC-1α: peroxisome proliferator-activated receptor γ coactivator 1α; SIRT3: sirtuin 3.

Previous studies demonstrated that AMPK may serve as a modulator of autophagy, thereby playing a protective role in IDD (Figure 3). Through the induction of autophagy, AMPK upregulates the ratio of Bcl-2/Bax and downregulates the level of cleaved caspase-3, so as to exert an anti-apoptotic role in TBHP-exposed rat NP cells (Zhang et al., 2018). Besides, AMPK-mediated autophagy also suppresses the increase in the expression of p21WAF1 and p16INKa, along with the upregulation in the phosphorylation of p53, and resists rat NP cell senescence induced by TBHP. Through modulating the expression of anabolic genes, including Col2A1 and Acan, as well as catabolic genes, including MMP-3, MMP-13, ADAMT-4 and ADAMT-5, autophagy induced by AMPK promotes the synthesis of ECM and inhibits the degradation of ECM, thus avoiding ECM destruction induced by TBHP (Chen et al., 2016; Kang et al., 2019). Several crucial proteins accounts for the regulation of AMPK in autophagy in the progression of IDD (Figure 3). In human NP cells exposed to TBHP, the activation of AMPK significantly reduces the phosphorylation of mammalian target of rapamycin (mTOR). The inactivation of mTOR further brings about the phosphorylation and activation of UNC-51-like kinase 1, which is a core protein in autophagy pathway possessing serine/threonine kinase activity. The activation of UNC-51-like kinase 1 subsequently leads to the assembly of autophagosome and then restores of blocked autophagic influx induced by TBHP (Kang et al., 2019). Apart from the illustrious mTOR, NAD-dependent deacetylase sirtuins (SIRTs) are also involved in the protection of AMPK against IDD. By upregulating the expression of SIRT1, AMPK induces autophagy and abates the over-expression of MMP3 in human NP cells stimulated by TNF-a (Wang et al., 2016). Besides, SIRT3 expression is also subject to the regulation of AMPK (Figure 3). By inducing the phosphorylation of peroxisome proliferator-activated receptor γ coactivator 1α (PGC-1α), AMPK increases the expression of SIRT3 in human NP cells. Through the regulation of SIRT3, AMPK further protects human NP cells against AGEs-induced mitochondrial apoptosis. Moreover, the upregulation of SIRT3 mediated by AMPK ameliorates AGEs-induced oxidative stress in human NP cells through inducing the expression of critical proteins in mitochondrial antioxidant networks, including superoxide dismutase 2, catalase, thioredoxin 2 and thioredoxin reductase 2 (Song et al., 2018). By the regulation of PGC-1α-SIRT3 pathway, AMPK also enhances mitophagy in rat NP cells. Mitophagy induced by AMPK eliminates damaged mitochondria to maintain mitochondrial homeostasis. Through the upregulation of mitochondrial antioxidation signals as well as the enhancement of mitochondrial dynamics and mitophagy, the activation of AMPK-PGC-1α-SIRT3 pathway suppresses mitochondrial apoptosis and senescence in rat NP cells exposed to TBHP (Wang et al., 2018). AMPK-induced activation of PGC-1α-SIRT3 pathway can also inhibit the secretion of pro-inflammatory cytokines, including IL-1β and IL-6, and relieve the imbalance between the synthesis and degradation of ECM in rat NP cells stimulated by oxidative damage (Lin et al., 2021). Acetyl-CoA carboxylase (ACC) is widely known as an important intermediate metabolite in fatty acid metabolism. By converting acetyl-CoA to malonyl-CoA, ACC plays an inhibitory role in the oxidation of free fatty acids. However, a recent study suggests ACC as a target of AMPK in lipopolysaccharide-stimulated rat NP cells (Figure 3). By inducing the phosphorylation of ACC, AMPK diminishes the production of free radicals, such as ROS and nitric oxide, as well as a series of pro-inflammatory cytokines, including IL-1β, IL-6 and TNF-α. Moreover, AMPK-mediated activation of ACC also retains the content of aggrecan and collagen II and suppresses the expression of major degradation enzymes for ECM, including MMP-3, MMP-13, ADAMTS-4 and ADAMTS-5 (Liu et al., 2019b).

In contrary to above protective role of AMPK, Yang and his colleagues reported the deleterious effect of AMPK on IVD (Figure 3). In degenerative rat IVD, the phosphorylation of AMPK is enhanced. Activation of AMPK further dephosphorylates mTOR. Inhibition of mTOR mediated by AMPK not only accelerates cell apoptosis and senescence, but also suppresses cell proliferation and cell cycle progression in degenerative rat NP cells (Yang et al., 2019). In addition to NP cells, AMPK also plays a role in the apoptosis of human AF cells. Through the inactivation of mTOR, AMPK decreases the ratio of Bcl-2/Bax and induces the release of mitochondrial cytochrome c, thereby activates caspases-dependent mitochondrial apoptosis. Besides, AMPK-mediated suppression of mTOR also promotes extrinsic apoptosis in degenerative AF cells through upregulating the expression of Fas and Fas ligand (Wu et al., 2017).

### Non-coding RNAs as AMP-Activated Protein Kinase Regulators in Intervertebral Disc Degeneration

Accumulating evidences demonstrated the regulatory role of ncRNAs in the activation of AMPK in IDD. Through targeting eukaryotic elongation factor 2, miR-143-5p induces the phosphorylation of AMPK. The activation of AMPK pathway mediated by miR-143-5p restricts cell proliferation and facilitates cell apoptosis, senescence and ECM degradation, so as to accelerate IDD progression (Yang et al., 2019). As one of the most studied long ncRNAs, HOX transcript antisense intergenic RNA (HOTAIR) was reported to regulate lipid accumulation in non-alcoholic fatty liver disease via the modulation of AMPK (Guo et al., 2021). In NP cells, HOTAIR acts as an AMPK regulator as well. By activating AMPK and then inactivating mTOR, HOTAIR promotes autophagy in rat NP cells. Moreover, autophagy mediated by HOTAIR further enhances cell apoptosis, senescence and ECM destruction, thus having a catalytic role in the course of IDD (Zhan et al., 2020).

## Targeting AMP-Activated Protein Kinase in the Treatment of Intervertebral Disc Degeneration

**FIGURE 4:**
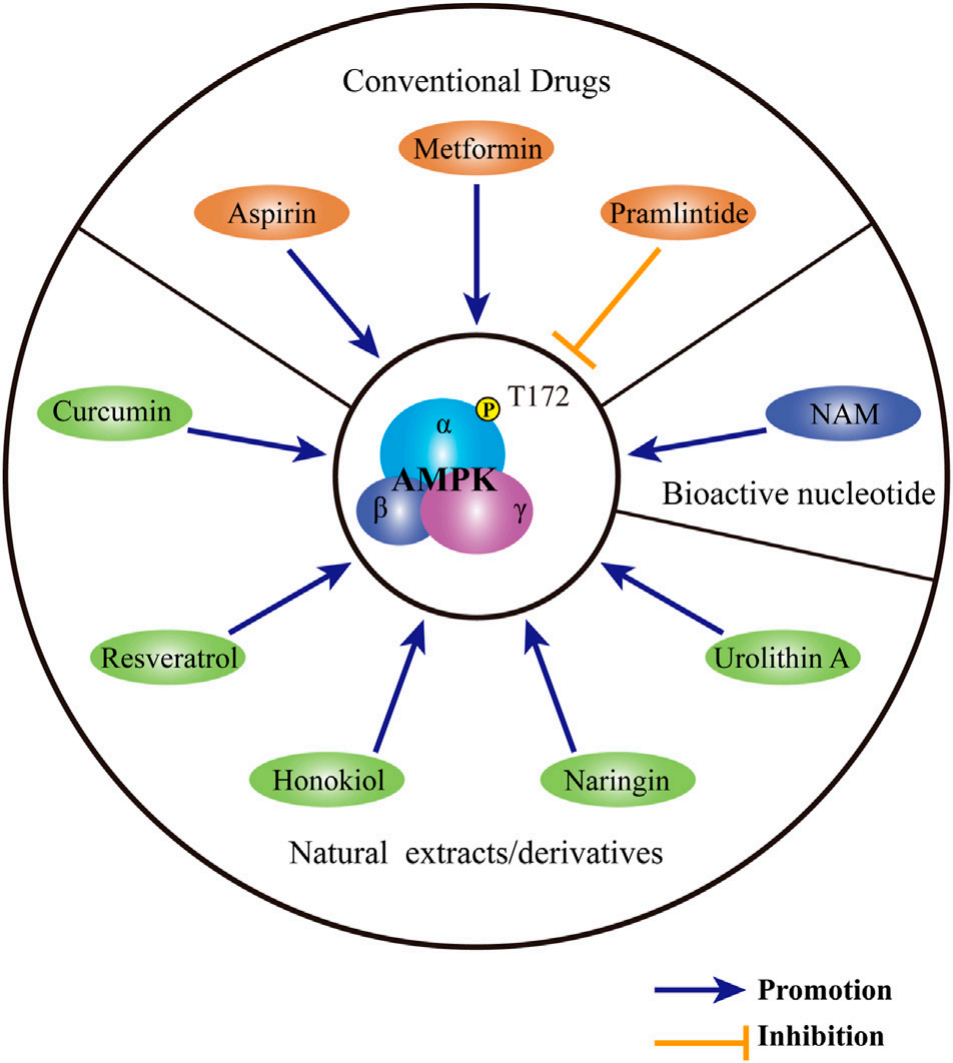
Substances targeting AMPK for the treatment of IDD. Conventional medicines, including metformin, pramlintide and aspirin, show therapeutic potential for IDD through regulating AMPK activity. A variety of natural extracts or derivatives, including curcumin, resveratrol, honokiol, naringin and urolithin A may activate AMPK to provide protection for IVD. As a bioactive nucleotide, NAM may ameliorate the progression of IDD by the modulation of AMPK. AMPK: AMP-activated protein kinase; IDD: intervertebral disc degeneration; NAM: nicotinamide mononucleotide.

Given the crucial role of AMPK in IDD, therapies targeting AMPK might be promising. To date, a wide range of compounds have been confirmed beneficial for the mitigation of IDD through the modulation of AMPK activity (Figure 4). As the most commonly used antidiabetic drug in clinic, metformin is identified as an AMPK agonist. By upregulating the phosphorylation of AMPK, metformin induces autophagy and protects rat NP cells against TBHP-induced apoptosis, senescence and ECM degradation (Chen et al., 2016). Akin to metformin, pramlintide also possesses both anti-hyperglycemic activity and protective ability for IDD. Specifically, pramlintide inhibits mitochondria-mediated apoptosis and promotes cell proliferation in human NP cells under hypoxic condition. Moreover, pramlintide also positively regulates anabolism but negatively regulates catabolism in human NP cells. The facilitation of pramlintide on cell survival and ECM metabolism might be attributed to its modulation on the activity of AMPK. However, pramlintide serves as an antagonist for AMPK. Through the inhibition of AMPK, pramlintide induces the phosphorylation of mTOR, thereby providing protection for human NP cells under hypoxia (Wu et al., 2018). Aspirin is a widely known non-steroidal anti-inflammatory drug. A recent study revealed the protection of aspirin against lipopolysaccharide-induced oxidative injury, inflammation and ECM destruction in rat NP cells. Besides, the effect of aspirin on IDD is dependent on its activation on the AMPK-ACC pathway (Liu et al., 2019b). The potential therapeutic effect of above three clinical drugs indicates the new use of conventional drugs in treating IDD.

Except for conventional drugs, natural extracts with a reputation for safety and accessibility, may also be beneficial for the treatment of IDD. As a prominent botanical extract with extensive and excellent biological effects, curcumin may serve as a preserver for IDD. By phosphorylating AMPK and activating the AMPK-mTOR- UNC-51-like kinase 1 pathway, curcumin induces autophagy in human NP cells. Autophagy induced by curcumin ameliorates apoptosis, senescence as well as ECM destruction induced by TBHP, thus providing protection for NP cells (Kang et al., 2019). Resveratrol is another phytoalexin with formidable anti-inflammatory and antioxidant effects. In human NP cells, resveratrol activates AMPK to upregulate the expression of SIRT1. Through increasing SIRT1 expression, resveratrol induces autophagy and suppresses the overexpression of MMP-3 in human NP cells induced by TNF-α (Wang et al., 2016). Similar to resveratrol, honokiol may also regulate the expression of sirtuin family members. Through the activation of AMPK-PGC-1α pathway, honokiol upregulates the protein level of SIRT3 and induces mitophagy in TBHP-treated rat NP cells. Through the maintenance of mitochondrial homeostasis, honokiol further relieves rat NP cell apoptosis and senescence and ameliorates rat IDD induced by acupuncture (Wang et al., 2018). As a major bioflavonoid derived from citrus, naringin activates autophagy in rat NP cells by phosphorylating AMPK. AMPK-dependent autophagy is held accountable for the protection of naringin against TBHP-induced apoptosis and ECM degradation in rat NP (Zhang et al., 2018). As a metabolite of ellagitannins produced by intestinal microflora, urolithin A can also induce mitophagy in an AMPK-dependent manner. By the enhancement of mitophagy, urolithin A protects rat NP cells against TBHP-induced mitochondrial apoptosis, thus playing an important role of defensive in IDD (Lin et al., 2020).

In addition to exogenous factors, endogenous regulators may also modulate the activity of AMPK to regulate the process of IDD. As a bioactive single nucleotide, nicotinamide mononucleotide (NAM) serves as the precursor for nicotinamide adenine dinucleotide. By synthesizing nicotinamide adenine dinucleotide, NAM participates in the generation of ATP. Through the regulation of cellular energy balance, NAM has anti-ageing ability and helps ward off a wide range of ageing-related diseases (Johnson et al., 2018; Kiss et al., 2019; Kiss et al., 2020; Miao et al., 2020). In human NP cells, NAM induces the phosphorylation of AMPK and activates downstream PGC-1α-SIRT3 pathway. Through the modulation of AMPK PGC-1α-SIRT3 pathway, NAM rescues human NP cells from AGEs-mediated oxidative injury and apoptosis. The protective effect of NAM is further verified in a rat model of IDD induced by acupuncture (Song et al., 2018).

## Discussion

As discussed above, the critical role of AMPK in IDD has been extensively investigated. However, reports concerning the activity of AMPK in IVD tissues during IDD are different. Moreover, although the majority of previous studies confirmed the protective role of AMPK in IDD, some just held the opposite opinion. We suppose that there might be dynamic changes in the activity of AMPK during the course of IDD. Besides, AMPK may play different roles at different stages of IDD. In endplate chondrocytes exposed to hydrogen peroxide, autophagy is enhanced at the initial stage. Then the level of autophagy decreases gradually. Besides, enhanced autophagy at the initial phase may protect endplate chondrocytes against oxidative damage (Chen K. et al., 2017). Similar beneficial effect of autophagy during early stage was also observed in NP cells under oxidative stress (Bai et al., 2020). As the upstream regulator of autophagy, AMPK may present dynamic changes similar to autophagy. We speculate that AMPK may be activated to provide protection in the initial stage of IDD. However, excessive stimulation beyond the protection of AMPK activation may impair the physiological capacity of IVD cells. Moreover, the activation of AMPK might be harmful at the late phase of IDD. If the hypothesis holds, AMPK-targeted therapies are supposed to be undertaken before the physiological function of IVD cells is impaired irreversibly. However, in order to verify this conjecture, further exploration is necessary.

It is widely known that developing new medications is time-consuming and labor-intensive. Therefore, new use of conventional drugs has attracted great interest in the past few years. So far, the protective effects of several old drugs, including metformin, aspirin and pramlintide has been preliminarily confirmed *in vitro* and in animal model of rat. Now that above drugs have been applied in clinical for several years, the safety of those drugs in suitable dosages may be exempt from evaluation. However, clinical trials are still required to assess the *in vivo* efficacy of above medicines on IDD. As mentioned above, DM serves as a predisposing factor for IDD. Therefore, DM and IDD may be comorbidity. Thus, metformin and pramlintide may exert anti-diabetic and IDD-protective effects at the same time. Aspirin is nowadays recommended for the prevention of cardiovascular events. Therefore, treatment of IDD patients with aspirin may not only postpone the progression of IDD, relieve discogenic or radicular pain, but also reduce risk of cardiovascular disorders. As an endogenous protective factor, the content of NAM is decreasing during ageing. Therefore, NAM supplement displays protective effect in multiple disorders associated with ageing including IDD. Now that NAM is ubiquitous in physiological conditions, exogenous supplementation of NAM is assumed to be safe. Nevertheless, the appropriate dosage of NAM for the alleviation of IDD remains further exploration. As for natural compounds, both the safety and validity *in vivo* are bound to be evaluated comprehensively. In addition to above small molecular compounds, ncRNAs are also engaged in the modulation of IDD progression via AMPK pathway. Currently, research focusing on the regulatory role of ncRNAs in the activation of AMPK during the course of IDD is relatively less. Even so, the involvement of ncRNAs in the setting of IDD indicates the therapeutic potential of the modulation of ncRNAs expression. Currently, novel techniques, such as CRISPER/Cas9, have been applied into the *in vivo* study of ncRNAs, which provides possibility for the modulation of AMPK activity and the treatment of IDD through RNA interference.

In addition to IDD itself, therapies targeting AMPK may also be effective in relieving IDD-related radiculopathy. Specifically, painful radiculopathy was triggered in a rat lumbar disc herniation model established by autologous NP transplantation. In that model, the phosphorylation of AMPK was suppressed in dorsal root ganglia sensory neurons. Activation of AMPK with metformin mitigated hyperalgesia in lumbar disc herniation-induced radiculopathy (Liu et al., 2019a). Similarly, the improvement of mechanical allodynia in the distal extremity of mice treated with acupuncture was achieved by metformin and 4-chloro-N-(2-(4-chlorobenzyl)-3-oxo-2,3-dihydro-1,2,4-thiadiazol-5-yl) benzamide (O304), a novel pan-AMPK activator (Das et al., 2019). Above studies indicate the dual regulatory role of AMPK-targeted therapies on both IDD and radiculopathy.

## Summary and Outlook

In summary, despite controversies on the activity and function of AMPK in IDD, AMPK participates in the pathogenesis of IDD. The regulation of AMPK in IDD progression is attributed to its modulation on a wide range of pivotal pathophysiological changes, including cell apoptosis, senescence, inflammation, oxidative damage, ECM destruction, etc. Up to now, multiple molecules targeting AMPK, including conventional medicines, natural extracts and endogenous bioactive nucleotide, have shown protective action in IDD. Besides, interfering the expression of ncRNAs to modulate AMPK activity provides a novel research direction for the treatment of IDD. Nevertheless, unremitting efforts should be devoted before the application of AMPK-targeted therapies for IDD into clinic.

## Abbreviations

ACC, Acetyl-CoA carboxylase; ADAMTS, a disintegrin-like and metalloprotease with thrombospondin type-1 motif; ADP, adenosine diphosphate; AF, annulus fibrosus; AGEs, advanced glycation end products; AMP, adenosine monophosphate; AMPK, AMP-activated protein kinase; ATP, adenosine triphosphate; CEP, cartilage endplate; DM, diabetes mellitus; ECM, extracellular matrix; HOTAIR, HOX transcript antisense intergenic RNA; IVD, intervertebral disc; IDD, intervertebral disc degeneration; IL-1β, interleukin-1β; LBP, Low back pain; mTOR, mammalian target of rapamycin; MMP, matrix metalloprotease; NAM, nicotinamide mononucleotide; NP, nucleus pulposus; O304, 4-chloro-N-[2-(4-chlorobenzyl)-3-oxo-2,3-dihydro-1,2,4-thiadiazol-5-yl] benzamide; PGC-1α, peroxisome proliferator-activated receptor γ coactivator 1α; ROS, reactive oxygen species; SIRT, sirtuin; TBHP, tert-Butyl hydroperoxide; TNF-α, tumor necrosis factor-α; Thr172, threonine 172 residue.
